# Directional Atherectomy for Critical Lower Limb Ischemia: A Retrospective Observational Study

**DOI:** 10.7759/cureus.70840

**Published:** 2024-10-04

**Authors:** Ana M Botero-Mora, Marlon Anaya-Martínez, William Ramírez-Herrán, Lina M Botero-Mora, Iván R Arismendi-Ortiz, Carlos M Ardila

**Affiliations:** 1 Department of Vascular Surgery, Fundación Santa Fe de Bogotá, Bogotá, COL; 2 Department of Vascular Surgery, Universidad de Antioquia, Medellín, COL; 3 Department of Vascular Surgery, Hospital Alma Mater de Antioquia, Medellín, COL; 4 Faculty of Dentistry, Department of Basic Sciences, Universidad de Antioquia, Medellin, COL

**Keywords:** arterial occlusive disease, cohort study, critical limb ischemia, directional atherectomy, limb salvage

## Abstract

Objective: Chronic arterial obstructive disease of the lower limbs is a significant global health issue. Directional atherectomy offers advantages in treating critical ischemia. The objective of this study is to determine the clinical outcomes of patients with critical ischemia who underwent directional atherectomy using the TurboHawkdevice.

Methods: A retrospective review was conducted at Alma Mater Hospital in Medellín, Colombia, on all medical records of patients with critical limb ischemia (Rutherford Classes V and VI) who underwent directional atherectomy for infra-inguinal arterial disease.

Results: A total of 42 atherectomies were performed in 41 patients, with 61% classified as Rutherford V and 24.4% as Rutherford VI. The average lesion length to be treated was 60 mm. Calcifications were found in 45.2% of cases, and predilatation angioplasty was used in 42.9% of cases. Atherectomy sites included femoropopliteal segment (40%), superficial femoral (29%), infrapopliteal vessels (14.2%), and more than one vessel in 50% of cases. Technical success was achieved in 78.6% of cases and procedural success in 97.6%, and the need for adjunctive conventional balloon angioplasty was 21.4%, while drug-coated balloon angioplasties were performed in 50% of limbs. The overall complication rate was 14.4%, with embolism at 4.8%, dissection at 4.8%, puncture at 2.4%, and perforation at 2.4%. The average follow-up duration was 12 months, and major amputations were required in 23.8% of cases. Improvement in ankle-brachial index to ≥0.1 was seen in 77% of limbs. Limb survival at 30 days was 85%, and at 90 days, it was 83%. The overall survival rate in the study was 79%.

Conclusions: Directional atherectomy is a safe alternative for managing critical ischemia in Rutherford V and VI patients.

## Introduction

Chronic arterial obstructive disease (CAOD) of the lower limbs affects 3-12% of the global population [1], with a health cost exceeding 4.7 million dollars per year [2-4]. In addition to peripheral vascular involvement, patients also exhibit multilevel arterial disease, ranging from 35% to 90% [3,4]. This results in poor health status and the presence of multiple comorbidities such as diabetes mellitus, coronary artery disease, and cerebrovascular disease, significantly increasing morbidity and mortality for surgical procedures and deteriorating patients' quality of life [5].

Open surgery with arterial bypass has been the standard management for CAOD in the femoropopliteal segments for many years [6], especially in lesions classified as C and D by the Transatlantic Inter-Society Consensus (TASC) with demonstrated results in the Balloon Angioplasty versus Surgical Intervention for Severe Limb Ischemia (BASIL) trial [7], where patients who underwent surgical revascularization and survived for more than two years showed clear benefits in terms of amputation-free survival and overall survival compared to the conventional balloon angioplasty group [7,8].

Due to the scarcity of available arterial conduits, surgical complications, re-intervention rates for arterial bypasses of up to 28%, and the high surgical risk associated with patients, endovascular therapy has seen a significant increase in use over the last decade [8]. This therapy is especially indicated in patients with femoropopliteal lesions classified as TASC A and B, in selected patients with high surgical risk (TASC C), and in the treatment of infrapopliteal lesions, with the goal of limb salvage [2].

Among the endovascular arsenal for infrainguinal disease treatment are conventional or drug-coated balloon angioplasty, the use of conventional stents, drug-eluting stents, and cryotherapy [9-11]. These devices aim to increase vascular lumen size by pushing the atheromatous plaque against the arterial wall, with permeabilities between 44% and 84% at 12 months [12-15].

Atherectomy theoretically offers advantages over other endovascular techniques, such as plaque volume reduction, decreased arterial barotrauma, and the ability to treat arterial segments at sites of tension and flexion. In the US market, there are six classified devices, including excisional, ablative, aspirational, rotational, and directional atherectomy, of which the only available option in our region is directional atherectomy with the TurboHawk^TM^ device (Medtronic/Covidien Plymouth, Minnesota, USA). Clinical trials evaluating the performance of these devices are scarce [11-13], with a clear predominance of claudicant patients (75%), femoropopliteal disease (80%), showing combined results, with an average primary patency rate at one year of 63.5% [12-15].

Currently, there are few studies in our region and in the global literature that demonstrate the behavior of these devices in patients with critical limb ischemia of the lower limbs (Rutherford Classification IV, V, and VI), primarily due to the considerable variability in endovascular options, patient heterogeneity, devices used, and the inclusion of claudicant subjects in available studies, 98% of whom will not progress in their arterial disease and will only require medical management [1].

It is essential to determine which patient populations benefit most from this therapy, not only due to the factors mentioned above but also considering procedure-associated complications and the high cost for healthcare systems. The objective of this study is to determine the clinical outcomes of patients with critical ischemia who underwent directional atherectomy using the TurboHawk^TM ^device.

## Materials and methods

A retrospective review was conducted at Alma Mater Hospital in Medellín, Colombia, on all medical records of patients with critical limb ischemia (Rutherford Classes V and VI) who underwent directional atherectomy for infra-inguinal arterial disease. The data were accessed for research purposes, and the authors had no access to information that could identify individual participants during or after data collection. Demographic characteristics, comorbidities, and variables related to the procedure, complications, and patient follow-up were assessed. Approval was obtained from the Institutional Ethics Committee of Alma Mater Hospital, Antioquia, Medellín, Colombia (Ref. No. 9/23/206).

Inclusion and exclusion criteria

This study included patients with critical lower limb ischemia (Rutherford Classification IV, V, and VI) who underwent directional atherectomy for infra-inguinal disease. Inclusion criteria consisted of patients with complete medical records and follow-up data. Exclusion criteria included patients with non-critical lower limb ischemia, alternative treatments without directional atherectomy, incomplete medical records, and contraindications to the procedure or adjunctive therapies.

The evaluated outcomes were as follows: (1) technical success, defined as residual stenosis of less than 30% after directional atherectomy without the need for adjunctive therapies; (2) procedural success, defined as residual stenosis of less than 30% after requiring additional adjunctive endovascular procedures following directional atherectomy; (3) hemodynamic success, defined as an increase in the ankle-brachial index (ABI) > 0.1 after the procedure; (4) 30-day all-cause mortality; (5) limb survival, defined as the absence of the need for amputation over the metatarsal region (supracondylar, infracondylar, and hip disarticulation) at 30 days and three months; (6) patient survival during the follow-up period; (7) the need for major or minor amputation; and (8) post-procedure ambulatory capacity.

Data were collected from electronic medical records and patient readmissions to the institution between August 2014 and December 2015 for each treated patient. Results of non-invasive vascular studies were extracted from the reports documented in the medical records, as well as anatomical and morphological characteristics from invasive studies (diagnostic angiographies), such as lesion length, vessels subjected to atherectomy, and the number of outflow vessels (infrapopliteal vessels). Missing data were acquired through the review of geographic studies by a single clinician to minimize inter-observer variability.

For follow-up, outpatient clinic records from the institution were evaluated, and direct telephone surveys with patients were conducted. Data entry was performed into a database designed using Google Drive® for accessibility by the researchers.

Intervention

All treated patients were presented at the medical board of the Vascular Surgery Department of the Clinic, where clinical conditions and procedure indications were evaluated with the participation of at least four specialists. Atherectomy procedures were performed by three vascular surgeons following the device's guidelines and institutional protocols for endovascular therapy, thereby reducing variability among operators. The intervention took place in a hybrid operating room, using fluoroscopy with a C-arm (Elite 9900, General Electric, Massachusetts, USA), under local anesthesia and sedation or general anesthesia, depending on the patient's comorbidities and requirements. The preferred inguinal approach was contralateral, and antegrade femoral access was used in patients with distal lesions of the popliteal artery and infrapopliteal vessels. Arterial puncture was performed using a micro-puncture set (MPIS) under ultrasound guidance, with an exchange for Terumo 8 Fr x 11 cm introducers or Destination® 8 Fr x 45 cm guide catheters, depending on the access site.

Patients were routinely heparinized to maintain an activated clotting time (ACT) between 200 and 300 seconds during the procedure. They were also antiplatelet loaded with a dose of acetylsalicylic acid (ASA) of 300 mg and clopidogrel 300 mg. Initial angiographic images were taken. A hydrophilic Terumo radiofocus® 0.035 Fr guide wire was advanced across the lesions. Then, a guide catheter (4 Fr Vertebral or multipurpose) was advanced, later exchanged for a Nitrex® 0.014 Fr Covidien guide wire, or for the distal embolic protection filter guide SPIDERFXTM. This was then positioned over the dominant infrapopliteal vessel or the distal popliteal artery, using it only in selected patients with chronic total occlusions, the presence of calcified plaque in the initial angiography, a single outflow vessel, and when the device was available in the institution, meeting the previous criteria.

Pre-dilation angioplasty (conventional Ivascular Oceanus® balloon of less than 3 millimeters with a pressure not exceeding 3 atmospheres) was only used in those lesions that did not allow passage of the guide catheter. The directional atherectomy device used was the TurboHawk^TM^ plaque excision system (Medtronic/Covidien Plymouth, Minnesota, USA). This atherectomy catheter consists of a flexible working channel that travels over a 0.014 Fr guide wire. At its distal end, it has a reservoir for mechanical plaque compression and a highly efficient blade, with a lithium battery in its proximal portion. The choice of device to use, based on size (for small or large caliber vessels), plaque type, reservoir, and the number of passes of the blade over the plaque, was determined by the vascular surgeon, depending on the arteriographic findings and the vessel caliber to be treated.

Adjuvant procedures, such as transluminal balloon angioplasty, were performed in the presence of residual stenoses greater than 30% after the passage of the atherotome. The choice between drug-eluting balloons (Ivascular Luminor® - drug-coated covering, Paclitaxel) or conventional balloons (Ivascular Oceanus®/Covidien-Evercross®, Nanocross®) was determined based on patient comorbidities, lesion characteristics, and the availability of drug-eluting balloons in the institution. Angioplasties were not performed after the passage of the device in patients with residual stenoses of less than 30%.

The intervention in vascular beds other than those targeted by atherectomy was determined in advance by the medical board and, in some cases, by the vascular surgeon during the procedure. The institutional protocol defined the use of stents in cases of residual stenoses greater than 50% after atherectomy and angioplasty, as well as in cases of flow-limiting dissections unresponsive to transluminal angioplasty. At the end of the procedure, control angiographic images were obtained to assess final patency and the presence of device-related complications, which were treated during the procedure and/or hospitalization according to the vascular surgery team's discretion, using open and/or endovascular approaches.

Decannulation of patients was performed in the operating room after measuring ACT <150 seconds. Hemostasis was achieved through compression of the inguinal region and immobilization of the leg for 12 hours. If an open approach was used, arterial suturing and layered closure were performed. The use of closure devices such as the Angio-Seal^TM^ (St. Jude Medical, Minnesota, USA) was left to the preference of the vascular surgeon.

Post-procedure management

Following the procedure, all patients received 100 mg of ASA daily, atorvastatin 40 mg/day indefinitely, and clopidogrel 75 mg/day for three months. In addition, they received weight-based anticoagulation doses with low-molecular-weight heparins every 12 hours for 48 hours. Twenty-four hours after the procedure, photoplethysmography or plethysmography, as clinically indicated, along with volume waveforms, measurement of segmental pressures, ankle-brachial index, and/or finger-brachial index, were ordered to assess the hemodynamic impact of the procedure. Patients were scheduled for follow-up appointments after discharge to assess their clinical conditions.

Statistical analysis

Statistical analysis was performed using IBM SPSS Statistics for Windows, Version 23.0 (IBM Corp., Armonk, NY). Frequency distributions were used to describe patient characteristics and clinical conditions for qualitative variables. In adition, summary statistics and measures of central tendency were used for quantitative variables, depending on their distribution.

For limb survival time, Kaplan-Meier analysis was conducted, and the average limb survival time was calculated along with 95% confidence intervals. In addition, a survival plot was generated for this variable and for patient survival.

This study was conducted in accordance with the principles outlined in the Declaration of Helsinki and subsequent amendments. The research protocol was carefully reviewed and approved by the Research Ethics Committee of the Hospital Alma Mater de Antioquia (206/2023), ensuring that the rights and welfare of all participants were protected. Moreover, the study adhered to the guidelines for good clinical practice and respected the autonomy and confidentiality of all participants.

## Results

Demographic and clinical variables

During the study period, a total of 42 directional atherectomy procedures with TurboHawk^TM^ were performed in 41 patients (24 females; 57%). The mean age was 71 years. A total of 64.3% of the patients belonged to the contributory social security system, while 35.7% were part of the subsidized system in the country. Thirty-one percent of the patients came from rural areas of the country and 56.1% belonged to low socioeconomic strata. Other demographic variables are described in Table 1.

**Table 1:** Baseline demographic and clinical characteristics of the study population * 1 corresponds to the lowest status and 6 to the highest. ** mean ±SD, median (min-max). *** mean ± SD

Characteristic (n = 41)		Number (%)
Sex	Male Female	18 (43.9%) 23 (56.1%)
Age	Years	72 (±10) 70 (50-91) **
Socioeconomic Status*	1	2 (4.9%)
	2	12 (29.3%)
	3	11 (26.8%)
	4	1 (2.4%)
	5	2 (4.9%)
	6	0 (0.0%)
	Rural	13 (31.7%)
Social security	Contributory	26 (63.4%)
	Subsidized	15 (36.6%)
Education level	Illiterate	1 (2.4%)
	Elementary school	23 (56.1%)
	High school	4 (9.8%)
	College university	3 (7.3%)
	No data	10 (24.4%)
Caregiver	Yes	38 (92.7%)
	Geriatric home	3 (7.3%)
Comorbidities	Arterial hypertension	33 (80.5%)
	Diabetes	28 (68.3%)
	Dyslipidemia	17 (41.5%)
	Smoker	13 (31.7%)
	Chronic kidney failure	10 (24.4%)
	Previous revascularizations	10 (24.4%)
	Coronary heart disease	7 (17.1%)
	Chronic obstructive pulmonary disease	5 (12.2%)
	Stroke	4 (9.8%)
	Carotid disease	2 (4,9%)
	Ejection fraction median	60 (±11) ***
Rutherford category	4	6 (14.6%)
	5	25 (61%)
	6	10 (24.4%)
Ankle-arm Index upon admission		0.61 ± 0.18 ***

The comorbidities found in the patients were hypertension (78.6%), diabetes mellitus (69%), dyslipidemia (43%), active smoking (31%), chronic kidney disease (26%), coronary artery disease (19%), chronic obstructive pulmonary disease (COPD) (11.9%), and stroke (10%). Twenty-three percent of the patients had a history of revascularization, of which 80% were performed endovascularly, 10% with open surgery, and 10% with hybrid therapy.

Among the clinical variables, carotid disease was found in 4.8% of the patients. The average ejection fraction was 62%. The clinical stage classification of the disease according to Rutherford was stage IV in 14.3%, stage V in 61.9%, and stage VI in 23.8% of the patients. The mean initial ankle-brachial index (ABI) was 0.61.

Variables related to the procedure

Table 2 summarizes the key procedure-related variables.

**Table 2:** Procedural characteristics and outcomes * Mean (±SD), median (min-max)

Variable	Feature	Number (%)
Approach type	Percutaneous	36 (85.7%)
	Open	6 (14.3%)
Calcification	Yes	19 (45.2%)
Outflow vessel	0	1 (2.4%)
	1	29 (69%)
	2	8 (19%)
	3	4 (9.5%)
Atherectomy vessels	Superficial femoral	12 (29%)
	Popliteal	7 (17%)
	Femoropopliteal	17 (40%)
	Tibiofibular trunk	2 (4.8%)
	Popliteal-trunk-posterior tibial	1 (2.3%)
	Superficial femoral-popliteal-anterior tibial	1 (2.3%)
	Superficial femoral-tibiofibular trunk	1 (2,3%)
	Trunk-anterior tibial	1 (2.3%)
	Infrapopliteal vessels	6 (14.2%)
	More than 1 blood vessel	21 (50.0%)
Largest lesion size		70 (±39), 60 (10-170) *
Predilatation angioplasty		18 (42.9%)
Conventional balloon angioplasty		9 (21.4%)
Medicated balloon angioplasty		21 (50%)
Stent		0 (0%)
SpiderFX		18 (42.9%)
Technical success		33 (78.6%)
Other additional procedures		15 (35.7%)
Procedure success		41 (97.6%)
Fluoroscopy time (minutes)		17 (±8), 15 (5-35) *
Contrast used (mL)		97 (±36), 100 (50-200) *

The procedures were all performed by three surgeons from the department, with 66.7% carried out by IRAO, 19% by EC, and 14.3% by WR. The chosen approach was percutaneous in 85.7% of cases and open in 14.3%. Calcified vessels were present in 45.2% of procedures. Sixty-nine percent of the intervened limbs had only one outflow vessel, 19% had two vessels, 9.5% had three vessels, and 2.4% had no outflow vessels (one patient). The mean size of the largest lesion was 69.41 mm (ranging from 10 mm to a maximum of 170 mm).

Pre-dilation angioplasty was required in 42.9% of procedures. A distal embolic protection filter (SpiderFXTm) was used in 57% of cases. Vessels subjected to atherectomy included more than one vessel in 50% of cases, femoro-popliteal segment in 40%, superficial femoral artery in 29%, popliteal artery in 17%, and infrapopliteal vessels in 14.2%, including procedures in the anterior and posterior tibial arteries and the tibioperoneal trunk in 4.8%. Technical success was achieved in 78.6% of cases. Conventional balloon angioplasty was required for stenoses greater than 30% in 21.4% of cases, and drug-eluting balloon angioplasties were performed in 50%. No patients required the use of outflow stents or stents to address associated complications. The mean fluoroscopy time was 17.41 minutes. The average contrast volume used was 96.6 ± 35 milliliters.

The procedure success rate was 97.6%. The failure was observed in one patient with a 90% stenosis of the superficial femoral artery, which, after directional atherectomy and drug-eluting balloon angioplasty persisted with a 30% stenosis. No further intervention was performed, and limb salvage was achieved after six months of follow-up. Additional procedures in other vascular beds were required in 35.7% of patients, of which 94% were endovascular and 6% were open procedures.

Complications

The complications encountered are summarized in Table 3.

**Table 3:** Periprocedural complications

Complications	Yes	6 (14.4%)
Type of complication	Arteriovenous fistula	2 (4.8%)
	Embolization	2 (4.8%)
	Dissection	2 (4.8%)
	Punction	1 (2.4%)
	Perforation	1 (2.4%)

Complications occurred in 14.3% of all procedures and included the following: distal embolism in 4.8% (n = 2), treated with embolectomy and femoropopliteal bypass to the third portion in one patient and a supracondylar amputation due to disease progression in the other case; dissection in 4.8% (n = 2), of which one patient received endovascular treatment with conventional balloon angioplasty and the other patient was not intervened as it was a flow-favorable dissection; complications associated with the puncture site in 2.4% (n = 1), with an infected inguinal hematoma that required open intervention with drainage and antibiotic therapy. Arteriovenous fistulas occurred in 4.8% (n=2) of the intervened limbs; one case was managed with endovascular treatment using endoluminal angioplasty, with resolution confirmed in the follow-up angiography. The other patient had a clinically palpable fistula on the first postoperative day, without hemodynamic impact, and it resolved during the outpatient evaluation.

There was one case (2.4%) of superficial femoral artery perforation, which was managed with conventional balloon angioplasty without further procedures, resulting in limb salvage during the study period.

In general, complications were resolved through open (33.3%, n = 2), endovascular (33.3%, n = 2), and hybrid (16.6%, n = 1), and no treatment was required in 16.6% (n = 1) of cases.

Follow-up

Table 4 shows the results related to patient follow-up.

**Table 4:** Follow-up outcomes and clinical status * Median ± SD (min-max)

Follow-up		Number (%)
Amputation	Yes	15 (35.7%)
Amputation level	Supracondylar	7 (16.7%)
	Infracondylar	3 (7.1%)
	Minor	5 (11.9%)
Ankle arm index	Improvement	30 (77%)
	Without changes	3 (7.7%)
	Decrease	6 (15.3%)
Ankle arm index	Delta	0.21± 0.22 (-0.30 to +0.59) *
Ambulation	Yes	24 (57.1%)
Status	Finalized	32 (76.2%)
	Deceased	9 (21.4%)
	Lost to follow-up	1 (2.4%)
Additional procedures	Yes	2 (4.8%)
Procedure type	Open	1 (2.4%)
	Hybrid	1 (2.4%)
	Endovascular	0 (0%)

The average follow-up for the studied patients was 12 months (minimum 5 and maximum 21). The loss to follow-up was 2.4% and pertained to a patient who was discharged from the hospital in less than 30 days, residing in a rural area, and could not be reached by phone. However, this patient did not have any readmissions or outpatient visits at the institution.

Overall, amputations were required in 35.7% (n = 15) of cases throughout the study period, including supracondylar amputation in 16.7%, infracondylar amputation in 7.1%, and minor amputation in 11.9% of the amputated limbs. The need for additional procedures other than atherectomy occurred in 4.8% (n = 2) of limbs, with a hybrid procedure in 2.4% (n = 1) and an open procedure in 2.4% (n = 1) of the intervened limbs.

**Figure 1:**
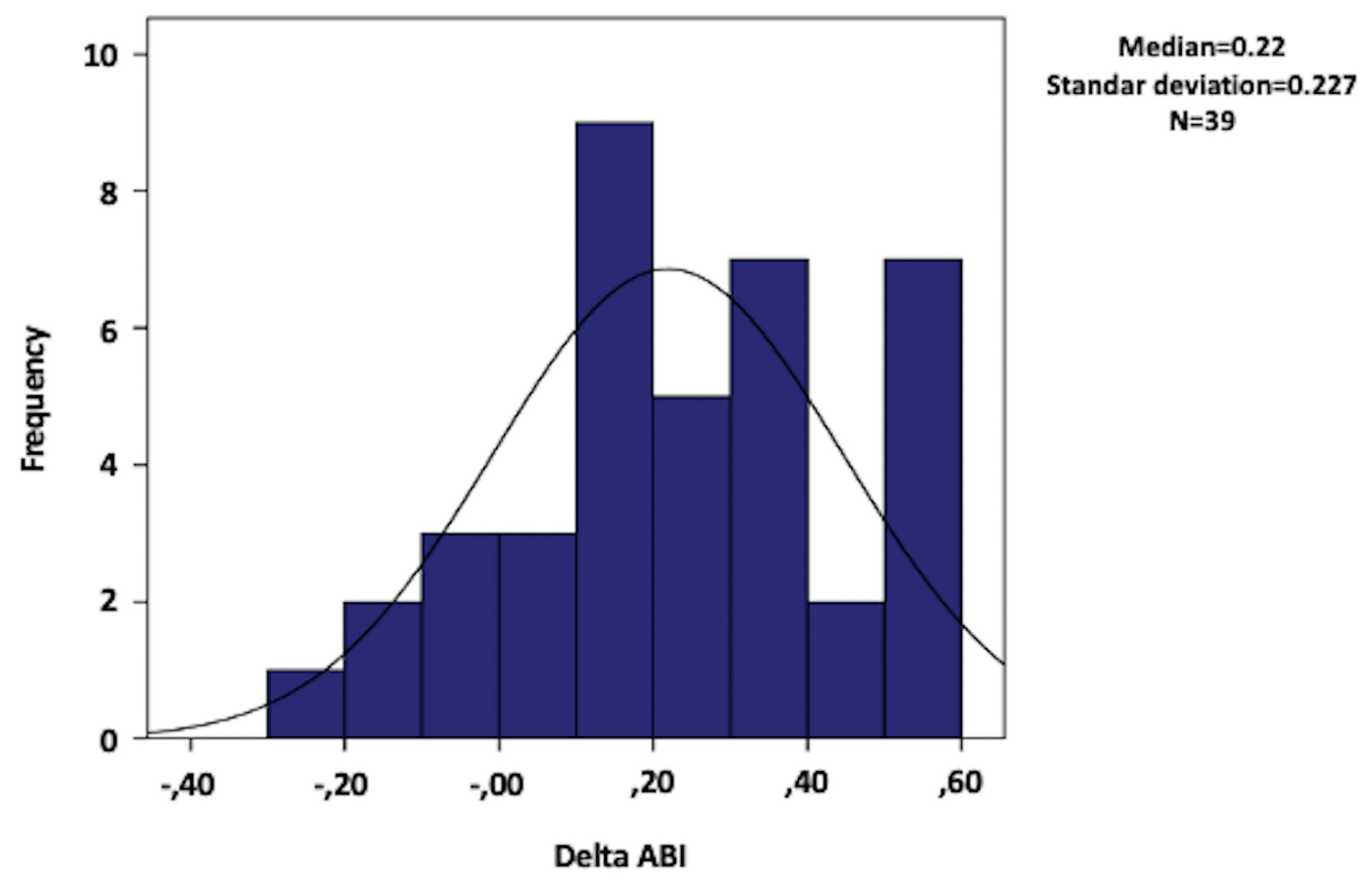
Ankle-brachial index (ABI) delta

For the ABI, a calculation of the change or delta in ABI from the pre-procedure value to the post-procedure value was performed, resulting in an average delta ABI of 0.21 (minimum -0.30 and maximum 0.59). Improvement in ABI was observed in 77% (n = 30) of the limbs, remained the same in 7.7% (n = 3), and decreased in 15.3% (n = 6). Among the six patients who experienced a decrease in ABI, two required major amputations, and two passed away; one was due to soft tissue sepsis in the leg where the atherectomy was performed and the other was due to heart failure. One patient experienced an embolism as a complication of atherectomy, but this patient had limb salvage at the end of the follow-up. Three limbs were lost to follow-up, with one patient not undergoing plethysmography, and two due to the loss of reports in the information system. Figure 1 displays a histogram illustrating the distribution of delta ABI.

**Figure 2:**
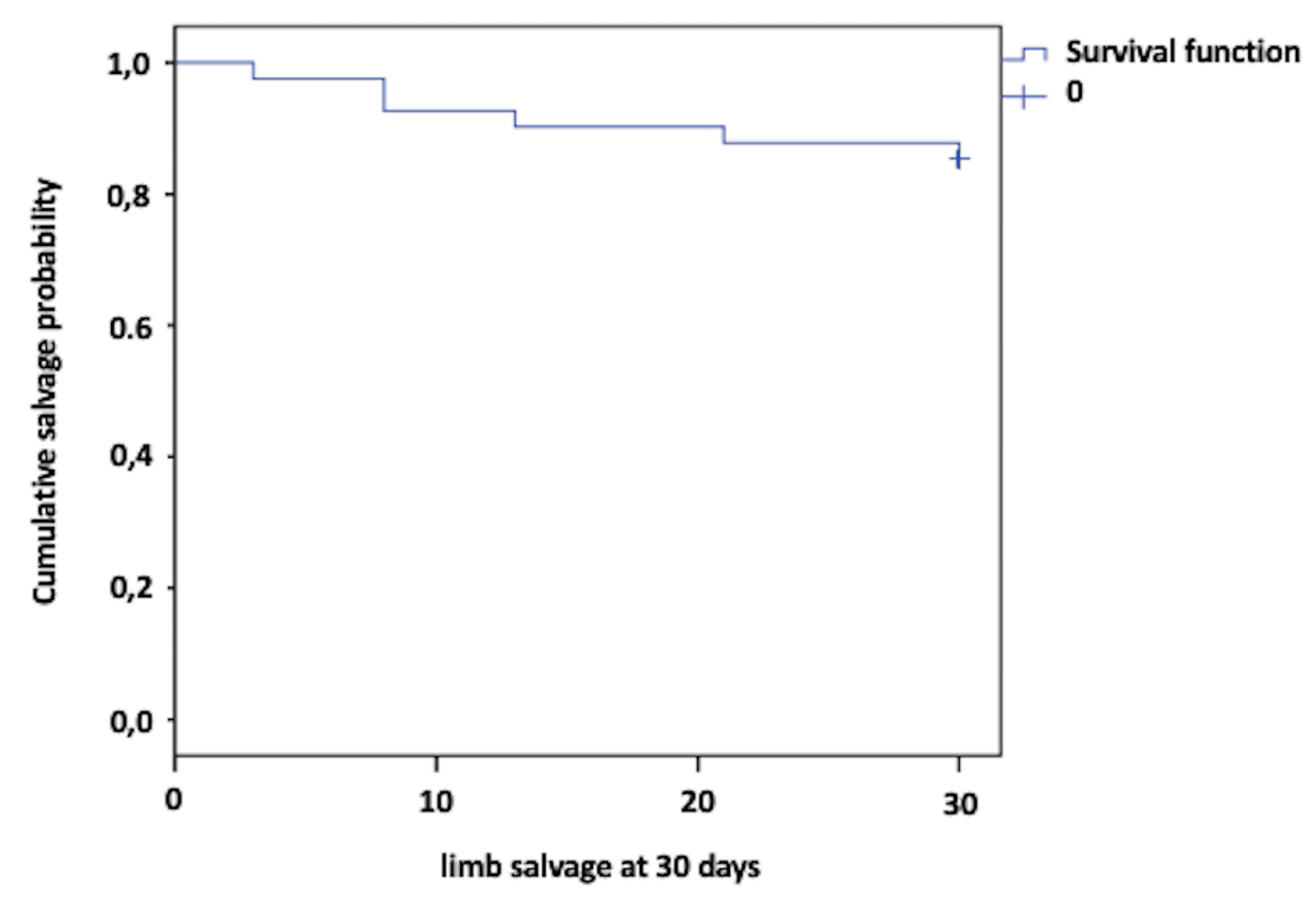
Kaplan Meier limb salvage curve at 30 days

Follow-up for limb salvage was conducted 30 days after the procedure, and it was observed that 85% of the limbs were functional, with an average survival time of 27 days. Six patients required amputation above the metatarsal level during this period (14.3%) (Figure 2).

**Figure 3:**
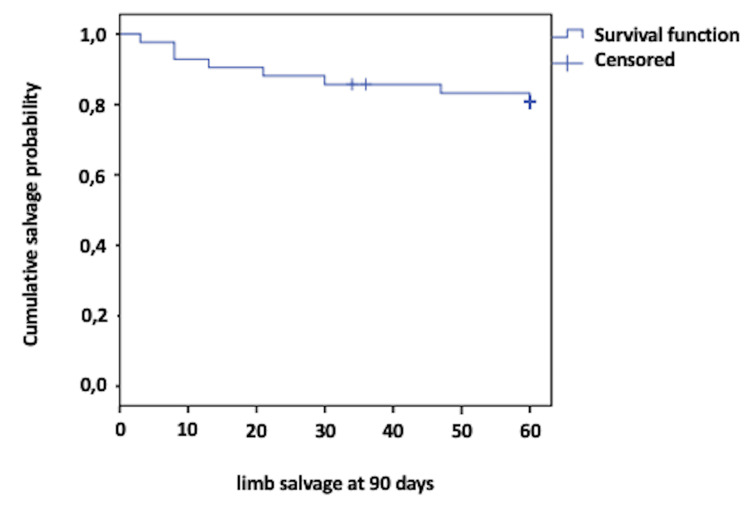
Kaplan-Meier limb salvage curve at 90 days

At the 90-day follow-up for limb salvage, 83% of the limbs were functional, with an average survival time of 53 days. Two additional major amputations were performed, bringing the total to 8.19% of the total limbs studied. Two more amputations occurred beyond 90 days (Figure 3).

**Figure 4:**
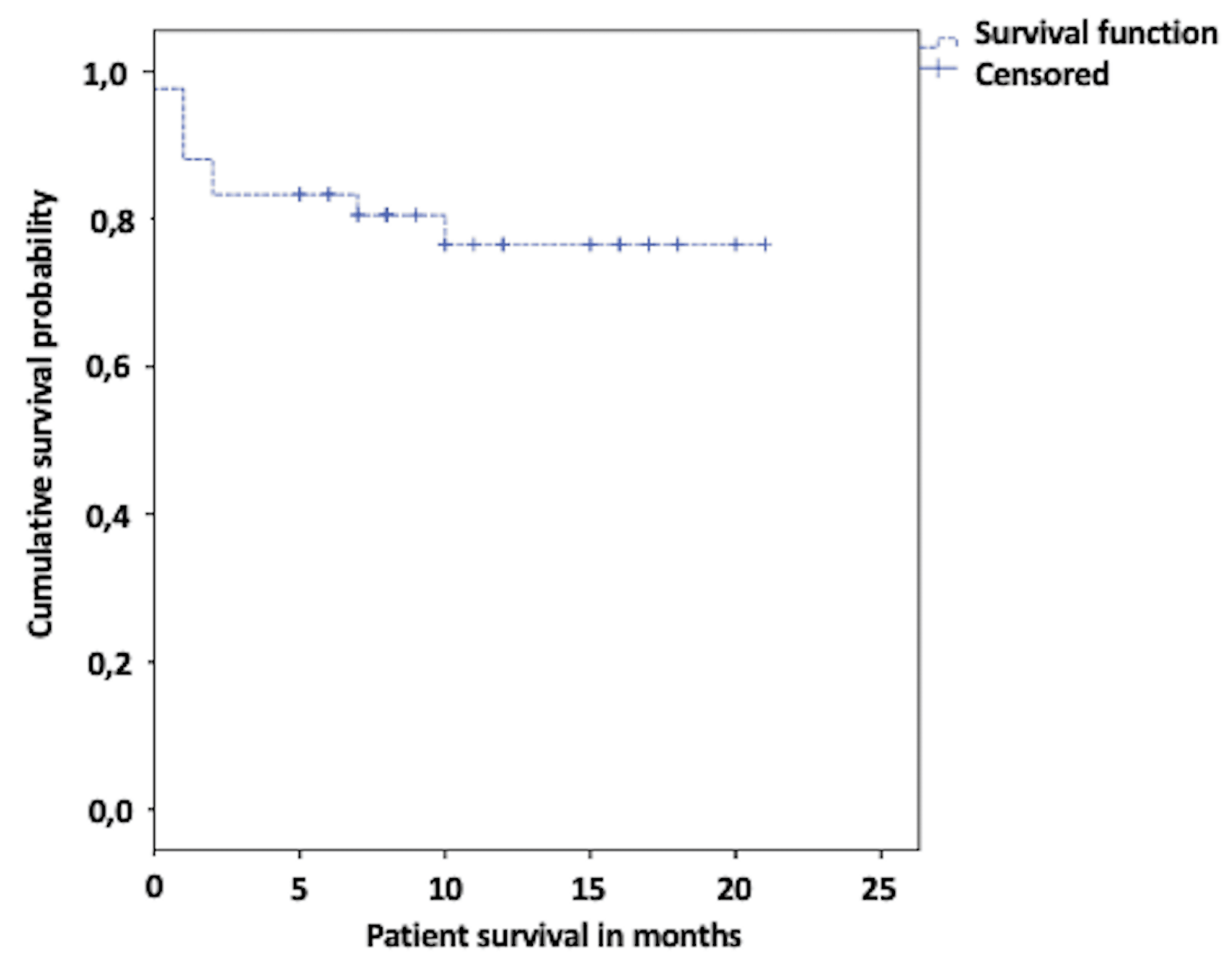
Kaplan-Meier survival during follow-up

Over the entire follow-up period, patient survival was 79%, with an average survival time of 16 months. There were nine deaths (21%) during the study, which included one case of soft tissue sepsis within the first 30 days (n = 1). Deaths occurring between 31 and 90 days were due to decompensated heart failure (n = 1), severe traumatic brain injury associated with status epilepticus (n = 1), soft tissue sepsis due to progression of arterial disease with a lack of acceptance of amputation upon readmission (n = 1), sacral and trochanteric pressure sore infection (n = 1), multi-lobar KPC pneumonia (n = 1), and urinary sepsis (n = 1). Beyond 90 days and until the end of the follow-up, deaths were due to a stroke (n = 1) and a patient with a hip fracture and end-stage renal failure (n = 1) (Figure 4).

## Discussion

Chronic occlusive arterial disease remains underdiagnosed, with a rising global incidence [3]. The use of endovascular procedures for the treatment of symptomatic patients has increased in frequency over the years, reaching up to 62% [16,17], especially in the infra-inguinal sectors, changing the paradigm of open surgery as the first management therapy [16,18]. Unfortunately, with this increase and the diversity of endovascular therapies, the scientific literature is scarce and controversial [19,20]. An example of this is the Cochrane Systematic Review [15], where no high-quality evidence was found to support the use of atherectomy over angioplasty in peripheral arterial disease. It should be noted that this review evaluated atherectomy devices with different mechanisms, and the authors reported studies with a high risk of bias.

In our study, we evaluated the clinical and demographic characteristics, limb salvage, and survival of 42 limbs and 41 patients with critical ischemia (Rutherford Classification IV, V, and VI) treated with the TurboHawk^TM ^system. Studies like DEFINITVELE [9] prospectively analyzed a subgroup of patients with critical ischemia (25% of the cohort). By contrast, studies like DEFINITIVE CA++ [12] and DEFINITIVE AR [13] included mostly claudicant patients or those with a less severe clinical spectrum of critical ischemia (Rutherford IV, rest pain), which accounted for only 15% in DEFINITIVE CA [12] and 43% and 36% in other studies [16,18]. Our case series included one of the highest percentages (85%) of patients with some tissue loss at study entry (Rutherford V and VI). These data were like those obtained by Kandzari et al. [21] but still higher for patients with Rutherford VI classification (24%), understanding that these patients have different clinical behaviors, as reported previously [22]. That study showed that perioperative mortality and one-year amputation-free survival are better for stage IV compared to Rutherford V and VI patients (93.6% vs. 78.3% and 87.7% vs. 66.7%), with this difference persisting for more than three years [22]. It has also been reported that patients may experience decreased functional status and often an inability to ambulate [21].

Among the comorbidities observed in the present study, the presence of diabetes stands out at 68%, which is comparable to previous reports (47-69%) [10-12,17-19]. Diabetes plays one of the most significant roles in the pathophysiology and natural history of chronic occlusive arterial disease (COAD), acting as both a risk factor and a trigger for complications. Sixty percent of non-traumatic lower limb amputations are attributed to this condition, and diabetic patients are 15% more likely to undergo amputations than non-diabetic individuals. Unfortunately, 85% of amputations in diabetics start with a lower limb ulcer. This is concerning due to its high prevalence in the general population. The World Health Organization estimated that in the year 2000, 3% of the global population had type I and II diabetes, amounting to 171 million people. This figure was projected for 2030, reaching 366 million, of which 80% will be diagnosed in developing countries [4].

The patients in the current study similarly represent other populations of critical ischemia patients, with a history of hypertension, dyslipidemia, chronic kidney disease, active smoking, and chronic obstructive pulmonary disease (COPD) [8-12,16-22], with the exception of coronary artery disease, which was found in 17% of our study population, only comparable to the results of the critical ischemia subgroup of DEFITIVELE [9].

Regarding the characteristics of angiographic lesions, there was no significant difference in lesion size compared to other reported data, with an average length of 70 mm, which is like that found in DEFINITIVELE [9] and longer than that observed in DEFINITIVE CA++ [12] and retrospective studies by Zeller et al. (39 and 43 mm) [23,24]. The presence of calcifications in our patients is one of the highest at 45%, exceeded only by studies like DEFINITIVE CA++ [12], which, due to its design and inclusion criteria, encompassed a group of COAD patients with femoropopliteal arterial calcifications who underwent directional atherectomy. Arterial calcification has previously been associated with a poor response to angioplasties, early recoil of treated lesions, and the need for stent placement due to flow-limiting dissections [14]. In prospective studies like DEFINITIVE AR [13] (DAART therapy), the use of drug-coated balloons after plaque excision with directional atherectomy allowed for primary patency rates as high as 90% at one year [14], compared to drug-coated balloon angioplasty as monotherapy, which only achieves primary patency rates of 78%. In addition, it eliminates the need for bailout stent placement, which can reduce one-year patency to 63-77% and introduce the disadvantage of leaving endoluminal material, making secondary interventions more complicated [10]. The use of drug-coated balloons in this study was 50%, and the need for bailout stent placement was 0%, which is comparable to DEFINITIVE AR [13] but lower than TALON (6.3%) [11], DEFINITIVE CA++ (4.8%) [12], DEFINITIVELE (2.0%) [9], and the results reported by Kandzari et al. (6%) [21].

Regarding variables associated with the procedure, it is important to highlight in our study the performance of infrapopliteal atherectomies in 14.2% of cases. This is because few authors have investigated the performance of directional atherectomy in these segments, and the device initially did not have approval for use in these vascular beds. Studies like TALON, with 24.4% of infrapopliteal atherectomies [11], and works like that of Tan et al. [24], with technical success rates of 90% and 96%, respectively, and limb salvage at six months of 81%, demonstrate the effectiveness and safety of directional atherectomy in this vascular territory.

The use of pre-dilation angioplasty was 42.9% of the intervened limbs, which correlates with the presence of severe stenoses and chronic total occlusions (CTOs), reported in a mean of 58% in atherectomy studies (26-63%) [10]. However, other studies only report the use of pre-dilation angioplasty between 7% [8] and 33% [23-25], possibly due to the availability of reentry devices, a greater variety of guide wires, and support catheters that allow for more effective lesion crossing. Technical success (78%), procedural success (97.6%), the need for adjuvant endovascular techniques (21.4%), contrast times, and fluoroscopy times correlate with what has been reported by other studies. It is important to emphasize that, in capable hands, this technique is reproducible [4,24].

The use of distal embolic protection devices such as SpiderFXTm was 42%, compared to the 22% to 60% described in other studies [23,24]. This can be explained by the presence of calcified lesions and a single outflow vessel in the initial angiography in 69% of cases. Studies like PROTECT [25,26] found major macroscopic debris larger than 2 mm in filters placed in patients undergoing atherectomy in 90.9% of cases. In our opinion, their use is clearly justified in patients with a single outflow vessel, although the value of distal embolic protection in atherectomy remains controversial [12,27,28].

Post-operative complications occurred in 14.3% of limbs overall, requiring treatment in 12% (4.8% endovascular, 4.8% hybrid, and 2.4% open surgery). This value is close to that seen in other studies [9,18,21]. In this study, the presence of two arteriovenous fistulas in 4.8% of limbs was reported, which has not been previously described for directional atherectomy. There was no hemodynamic impact, and these patients were treated with endovascular therapy using conventional balloon angioplasty, achieving intraoperative resolution in one patient and within the first 30 days in the other. One of them died 10 months later due to chronic renal failure, but both retained their limbs. Arterial perforation, which is the most frequent complication in studies like DEFINITVELE (5.3%) [9] but reported as absent in studies published by Zeller et al. [23], Schwartz et al. [8], and Yancey et al. [29], occurred in our study in 2.4% of limbs. It was treated with conventional balloon angioplasty, resulting in resolution and limb salvage for the patient during follow-up.

In this study, distal embolism occurred in 4.8%, which is lower than that reported in the DEFINITIVELE cohort of critical ischemia patients [17] and in other studies by Kandzari et al. [21], Shammas et al. [25], and Schwartz et al. [8], which reported rates of 7.5%, 7%, 5%, and 5%, respectively. However, it is higher than the records from the TALON study [11], where embolism occurred in only 0.1% of the intervened patients, and the DEFINITIVE CA++ study [12], which reported only a 2.5% incidence. This difference can be explained by the presence of total occlusions and the need for pre-dilation angioplasty, which can result in embolization rates of up to 30% [19]. The incidence of dissection and complications associated with arterial puncture did not differ from what has been previously reported.

In this study, the follow-up outcomes were based on limb survival rates at 30 and 90 days, overall patient survival, ambulation capability at the end of follow-up, and, as an early outcome, changes in the ankle-brachial index (ABI) from baseline compared to post-procedure values. Ultrasounds were not performed as part of the protocol to assess primary and secondary patency of the intervened vessels, which limits the comparison with results from other studies but provides data on the hemodynamic impact of atherectomy. Improvement in the ankle-brachial index by >0.1 was observed in 77%, like the 80% improvement reported by Zeller et al. [23], DEFINITIVELE [9], and as demonstrated by Tan et al. [24], with delta ABI changes of 0.28, compared to those found in the patient group treated in the current study (0.21).

One-year follow-up analyses were not reported because 50% of the patients did not complete it. In our case, the major amputation rate was 23.8%, which is higher than the rates reported by Kandzari et al. [21], Zeller et al. [23], and Schwartz et al. [8]. This difference can be explained by the higher proportion of patients with Rutherford stage VI included in our study.

In the present study, limb salvage rates at 30 days were 85%, and at 90 days, they were 83%. Kandzari et al. [21], in a prospective study of 78 patients undergoing directional atherectomy for critical ischemia, reported limb salvage rates at 30 days of 92% and at six months of 82%, which were like other studies such as that by Schwartz et al. [8]. The difference between these reports can be explained because limb salvage data were estimated without discriminating the Rutherford stage, and the results were better due to the inclusion of claudicant patients, as seen in the cohort of McKinsey et al. (95%) [9]. The ambulation capability of our patients over the follow-up period was sustained at only 57.1%, which is low. However, this information is consistent with other studies, which correlate this outcome with patient comorbidities, the severity of arterial disease, and systemic involvement affecting vascular beds at multiple levels, thereby impairing their quality of life [21,27].

Although in some cases amputations in critical ischemia patients are not avoidable, there is a need to find alternative interventions that reduce the risk of complications aimed at limb preservation. The BASIL trial demonstrated the superiority of arterial bypass in patients with severe peripheral arterial disease compared to conventional balloon angioplasty. However, since its introduction, improvements in patency rates have been described for other therapies, such as directional atherectomy, which offers benefits with lower perioperative morbidity and mortality. Patients with critical ischemia belong to a spectrum of the disease with greater severity and worse outcomes. Therefore, these patients should be offered procedures that mitigate surgical and percutaneous intervention risks.

The findings of the BEST-CLI study, published in 2022 [28], conclude that in patients with an available autologous saphenous graft suitable for surgical revascularization, surgical management as a primary strategy reduces the risk of death or adverse limb events by 32%. However, in patients without the availability of a saphenous graft, no statistically significant difference was demonstrated between both interventions. Approximately 7-8% of patients in both cohorts underwent atherectomy as endovascular therapy, with uncovered stents and conventional angioplasty being the most used strategies. It is noteworthy that, like our study, cases of atherectomy were reported in both the femoropopliteal and infrapopliteal segments. Technical success rates of endovascular therapy were comparable in our study (78% vs. 84.7% and 80.6% in both BEST-CLI cohorts, respectively). The rate of supracondylar amputations in our study was 16.7%, like the 14% reported in the endovascular therapy arms of both BEST-CLI cohorts. While some outcomes are comparable, specific outcomes within the subgroup of interest in the endovascular therapy group remain unknown. Nevertheless, in patients not eligible for primary surgical management due to the unavailability of autologous saphenous grafts or those at high surgical risk, our study suggests directional atherectomy as part of the endovascular management arsenal, yielding results like those reported in the literature.

Limitations of the study

The retrospective design of the present study prevents establishing a direct causal relationship, considered a limitation of studies with this design. The patients included in the study had multi-level diseases with a high likelihood of requiring major amputations. Therefore, strict inclusion criteria are needed for limb salvage outcomes to define the subgroup of patients who derive the greatest benefits from directional atherectomy. For comparing results with other previously published studies, patient follow-up with ultrasound is necessary to assess patency rates over time and objectively evaluate the impact of the intervention. Another limitation of the current study is that it did not systematically compare differences between open and endovascular modalities, so these results should preferably be confirmed with clinical trials.

## Conclusions

Directional atherectomy is a safe alternative for managing critical ischemia in Rutherford V and VI patients, with known complications that can be mostly managed with adjunctive endovascular therapy. While its cost may be higher, its impact on limb and patient survival is fully justified. Prospective studies are needed to compare the effectiveness of this technique with open surgery and other endovascular techniques, specifically in this subgroup of patients, where limb salvage, quality of life impact, and ambulation capacity are the most important outcomes.
